# Protective Effect of Mitochondrial ND2 C5178A Gene Mutation on Cell and Mitochondrial Functions

**DOI:** 10.1155/2021/4728714

**Published:** 2021-07-20

**Authors:** Liuyang Tian, Chao Zhu, Huanwan Yang, Yang Li, Yuqi Liu

**Affiliations:** ^1^School of Medicine, Nankai University, Tianjin 300071, China; ^2^Cardiac Department, The Institute of Geriatric Cardiology PLA General Hospital, Beijing 100853, China; ^3^Department of Cardiology, Beijing Friendship Hospital, Capital Medical University, Beijing 100050, China; ^4^Department of Cardiology & National Clinical Research Center of Geriatrics Disease, Chinese PLA General Hospital, Beijing 100853, China; ^5^Beijing Key Laboratory of Chronic Heart Failure Precision Medicine, Chinese PLA General Hospital, Beijing 100853, China; ^6^National Key Laboratory of Kidney Diseases, Chinese PLA General Hospital, Beijing 100853, China

## Abstract

**Background:**

Mitochondrial NADH dehydrogenase subunit 2 (MT-ND2) m. 5178C>A gene mutation has protective effects against various diseases, but the molecular mechanism is still unclear. In previous study, we found a heteroplasmy level of MT-ND2 m. 5178C>A mutation in normotensive controls. Peripheral blood samples were obtained from essential hypertension individuals carrying the mutation and healthy controls without gene mutation to establish immortalized lymphocyte lines. To investigate the effect of the MT-ND2 m. 5178C>A gene mutation, comparative analyses of the two group cell lines were performed, including measurements of cell proliferation, viability, ATP synthesis, mitochondrial oxidative stress, and oxidative phosphorylation.

**Results:**

The cell proliferation rate and viability of the MT-ND2 m. 5178C>A mutant lymphocyte line were higher than those of the control group. Mitochondrial functions of the MT-ND2 m. 5178C>A mutant lymphocyte were increased, including increased ATP synthesis, decreased ROS production, increased mitochondrial membrane potential and Bcl-2 gene transcription and protein translation, decreased Caspase 3/7 activity, and decreased early apoptosis and late apoptosis. The oxygen consumption rate (OCR) of the mutant lymphocyte line was higher than that of the control group, including basal OCR, ATP-linked OCR, maximal OCR, proton leak OCR, and reserve OCR, and there was no significant difference in nonmitochondrial OCR. The activity of Mitochondrial Complex I of the mutant group was increased than that of the control group.

**Conclusions:**

The MT-ND2 m. 5178C>A mutation is a protective mutation that may be related to improvement of mitochondrial functions and decrease in apoptosis.

## 1. Background

Mitochondrial DNA (mtDNA) mutations have been reported in many diseases such as Leber hereditary optic neuropathy [1], maternally inherited deafness, aminoglycoside-induced Deafness [2], mitochondrial encephalomyopathy, lactic acidosis, and stroke-like episodes [3]. Mitochondria are the main site of oxidative phosphorylation (OXPHOS) in eukaryotic cells. Each cell contains 100 to 1000 mitochondria, each containing an independently encoded double-stranded circular DNA. Human (mammalian) mtDNA has 16 569 base pairs with 37 coding genes, including 22 mitochondrial transport RNA (tRNA) genes, two ribosomal RNA (12r RNA and 16r RNA) genes, and 13 peptides associated with the OXPHOS respiratory chain complex [4]. Mitochondrial dysfunction has been proved to be associated with many common human diseases, including diabetes, coronary heart disease, and cancer; neurodegenerative changes; and aging [5] OXPHOS contains approximately 90 proteins, and there is a close association between genes that transcribe and translate OXPHOS-related proteins, including nuclear and mitochondrial genes, to maintain and regulate the metabolic processes [6]. Further research found that the gene defect encoding OXPHOS protein directly leads to impaired OXPHOS function, further leading to a series of problems such as metabolic disorders, and is one of the important causes of various diseases [7]. In our previous study, we found that there is a heteroplasmy level of m. 5178C>A mutation between essential hypertension (EH) and normotensive controls. The heteroplasmy level of MT-ND2 m. 5178C>A in EH patients is much lower than that in control subjects. The MT-ND2 m. 5178C>A polymorphism of NADH dehydrogenase subunit 2 (ND2) 237Leu/Met was first reported to be associated with longevity [8]. Recently, the MT-ND2 m. 5178C>A mutation has been found to have protective effects in a variety of diseases such as myocardial infarction [9], cerebrovascular disease [10], type 2 diabetes [11], and atherosclerosis [12]. However, the mechanism of how MT-ND2 m. 5178C>A mutation associated with lots of diseases has not been elucidated. Therefore, we enrolled five cases from the EH group without MT-ND2 m. 5178C>A gene mutation and control groups with m. 5178C>A gene mutation. The gender, age, body mass index, and laboratory indexes were matched in two groups. Lymphoblastoid cell lines were immortalized by transformation with Epstein-Barr virus from the peripheral whole blood of each participant. Then, we investigated the mechanisms of the MT-ND2 m. 5178C>A gene mutation in cell and mitochondrial functions of lymphocyte lines to explain the protective effect of MT-ND2 m. 5178C>A gene mutation in the mechanism against EH.

## 2. Results

### 2.1. Clinical Characteristics

Basic clinical characteristics were compared between mutant and control groups. Triglyceride, SBP, and DBP were lower in the mutant group than in the control group (Table 1).

### 2.2. Lymphocyte Line Viability and Proliferation

**Figure 1:**
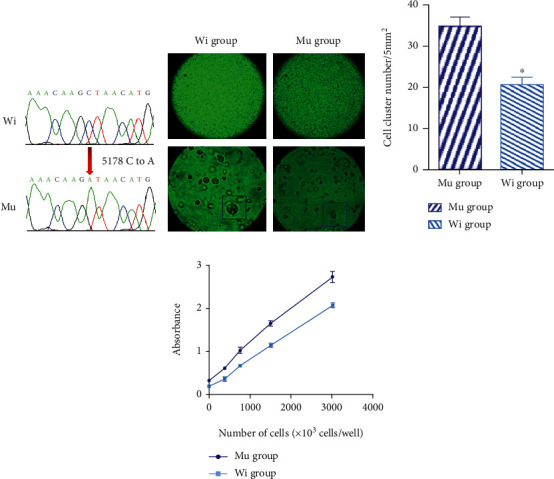
Cell activity and proliferation. (a) Partial sequence chromatograms with mutation at the ND2 5718 site. (b) The proliferation of the two groups of immortalized lymphocyte lines. (c) Comparison of cell proliferation in mutant and control cell lines. Proliferation of the mutant group was significantly higher than that of the control group. (d) Comparison of cell activity in mutant and control cell lines. CCK-8 was used to compare the cell viability of mutant and nonmutated lymphocyte lines. At the same cell number, the activity of the mutant group was higher than that of the control group. (^∗^*P* < 0.05).

Mitochondrial gene sequence analyses revealed that the mutation group carried the MT-ND2 m. 5178C>A gene mutation (Figure 1(a)). A previous study showed that this site mutation leads to amino acid substitution in the ND2-encoding gene from leucine (Leu) to methionine (Met) [8].

To further investigate whether ND2 gene mutations affect cell proliferation, we used the Epstein-Barr virus to infect lymphocytes.  Figure 1(b) shows the proliferation of the two groups after infection. The nucleuses of the mutant lymphocytes were more obvious under high magnification, and the cell membrane boundaries were blurry (Figure 1(b)). Proliferation of the mutant group was significantly higher than that of the control group (34.67 ± 4.16 vs. 20.67 ± 3.06, *P* = 0.009, Figure 1(c)).

### 2.3. Lymphocyte Line Proliferation

CCK-8 was used to compare the cell viability of mutant and nonmutated lymphocyte lines. Luciferin, the nonluminescent peptide, was transferred to luminescent luciferase and ATP under the action of Caspase 3/7, and the value of Caspase 3/7 is recorded by a Centro LB90 microplate luminometer. The activities of the mutant cells and control group were linearly related to the number of cells. At the same cell number, the activity of the mutant group was higher than that of the control group (*P* < 0.05, Figure 1(d)).

### 2.4. Lymphocyte Line ATP Synthesis

**Figure 2:**
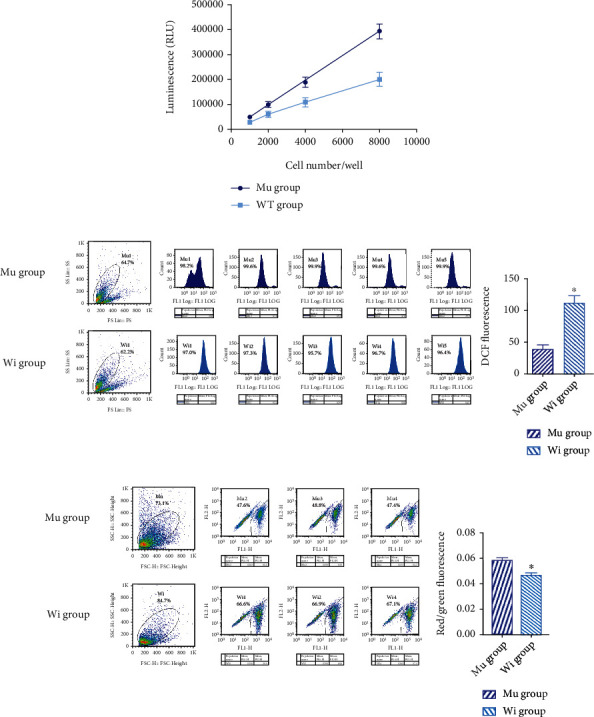
Mitochondrial functions of mutant and control cell lines. (a) ATP synthesis: ATP production was measured by a luciferin/luciferase luminescence assay. The ATP production of the mutant cells was significantly higher than the control group. (b) ROS synthesis: ROS production was detected through fluorescent probe DCFH-DA by flow cytometry. ROS produced by the mutant cell line was significantly lower than that in the control group. (c) Mitochondrial membrane potential: mitochondrial membrane potential reflects the functional metabolic status of mitochondria which is calculated by the ratio of red/green fluorescence. The value of the mutant cell line was significantly higher than control group. (^∗^*P* < 0.05).

Mitochondria are a cell's powerpacks. To determine whether mitochondrial gene mutation affects the ability of oxidative phosphorylation (OXPHOS) to synthesize ATP, we measured ATP production by a luciferin/luciferase luminescence assay. There was a linear correlation between ATP production and the cell number in both mutant and control cell lines. ATP production of the mutant cells was significantly higher than that of the control group (*P* < 0.05, Figure 2(a)).

### 2.5. Lymphocyte Line ROS Synthesis

Mitochondrial oxidative stress damage is the most important mechanisms of which mitochondria regulate cell apoptosis. In our study, we measure the intracellular ROS production through fluorescent probe DCFH-DA. The ROS production in the mutant cell line was significantly lower than that in the control cell line (39 ± 13.79 vs. 112.66 ± 23.13, *P* = 0.001, Figure 2(b)).

### 2.6. Mitochondrial Membrane Potential

Mitochondrial membrane potential (*ΔΨ*_m_) means the proton electrochemical gradient of mitochondrial membrane, which is reflective of the functional metabolic status of mitochondria [13]. This assay uses the ratio of red/green fluorescence of JC-10 Ex/Em = 540/590 and 490/525 nm (FL590/FL525) to reflect *ΔΨ*_m_. The *ΔΨ*_m_ value of the mutant cell line was significantly higher than that of the control group (0.0587 ± 0.0187 vs. 0.0469 ± 0.0165, *P* = 0.001, Figure 2(c)).

### 2.7. Apoptosis Protein Caspase 3/7 Activity

**Figure 3:**
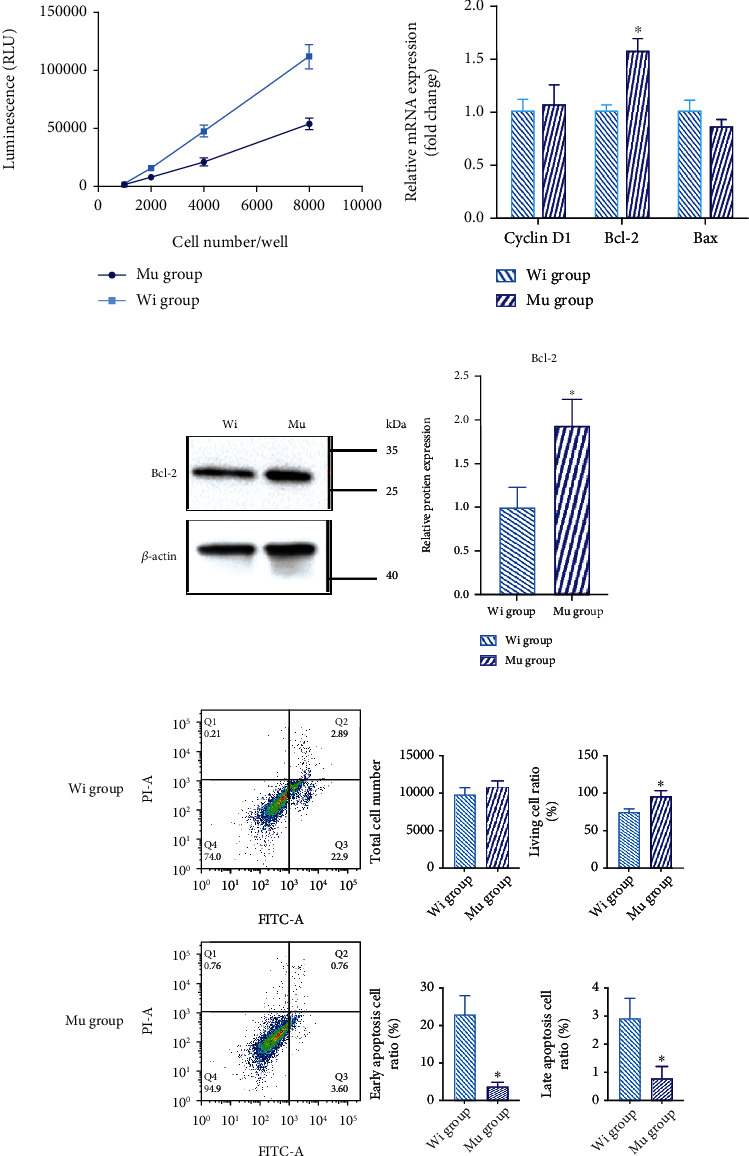
Proliferation and apoptosis of mutant and control cell lines. (a) Caspase 3/7 activity: Caspase 3/7 is a specific molecular marker of apoptosis. The expression level of Caspase 3/7 in the mutant group was significantly lower than that in the control group. (b) The transcription of proliferation and apoptosis genes: the mutant group showed increased Bcl-2 gene transcription compared with the control group. But there was no significant difference between Bax and Cyclin D1 gene transcription. (c) The protein expression of proliferation and apoptosis genes: the mutant cells showed increased Bcl-2 protein level. (d) Apoptosis assay: the apoptosis rate was detected by the FITC Annexin V Apoptosis Detection Kit. The living cell ratio of the mutant group was higher than that of the control group. The early apoptosis and late apoptosis in the mutant group are significantly decreased than those in the control group. (^∗^*P* < 0.05).

Another important function of mitochondria is to regulate cell apoptosis [14]. Caspase 3/7 is a specific molecular marker of apoptosis [15]. There was a linear correlation between Caspase 3/7 expression and the cell number in both mutant and control cells. At the same cell number, the expression level of Caspase 3/7 in the mutant group was significantly lower than that in the control group (*P* < 0.05, Figure 3(a)).

### 2.8. The Expression of Proliferation and Apoptosis Genes and Proteins

To evaluate the mechanism implicated in the MT-ND2 m. 5178C>A gene mutation decreased apoptosis, we measured the genes of apoptotic signal pathway through transcription and the protein expression. The mutant group showed increased Bcl-2 gene transcription compared with control group (1.58 ± 0.063 vs. 1.00 ± 0.036%, *P* = 0.0014) (Figure 3(b)). And mutant cells also showed increased Bcl-2 protein level than the control group (Figure 3(c)).

### 2.9. Apoptosis Assay

The apoptosis rate was detected by the FITC Annexin V Apoptosis Detection Kit. Cells that are in early apoptosis are FITC Annexin V positive and PI negative, and cells that are in late apoptosis are both Annexin V and PI positive. The living cell ratio of the mutant group was higher than that of the control group (94.9 ± 4.9% vs. 74 ± 3.20%, *P* = 0.0229, Figure 3(d)) when the total cell number was almost the same. The early apoptosis and late apoptosis in the mutant group are significant decreased than those in the control group (3.6 ± 0.75% vs. 22.9 ± 3.00%, *P* = 0.0229; 0.76 ± 0.27% vs. 2.89 ± 0.42%, *P* = 0.0125, Figure 3(d)).

### 2.10. Mitochondrial OCR Assay

**Figure 4:**
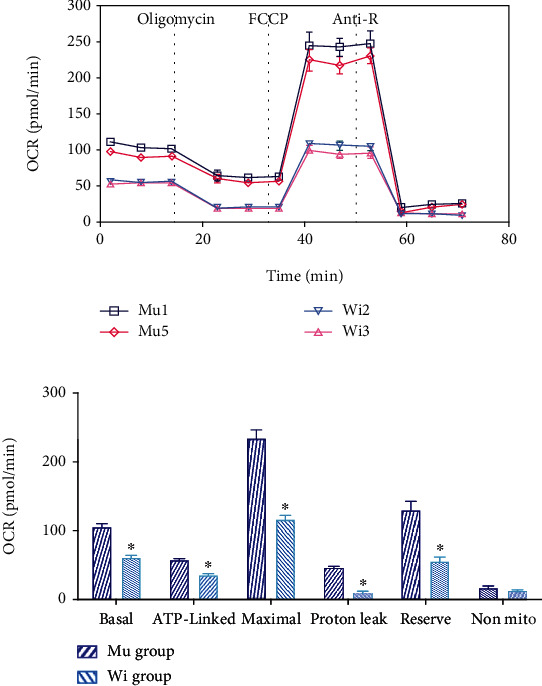
Detection of mitochondrial OCR in mutant and control cell lines. (a) OCR curves of mutant and control cell lines after treatment with various inhibitors. The compounds (oligomycin, FCCP, and a mix of rotenone and antimycin A) are serially injected to measure ATP production, maximal respiration, and nonmitochondrial respiration, respectively. Proton leak and spare respiratory capacity are then calculated using these parameters and basal respiration. (b) Statistical analysis of the basic OCR, ATP-coupled OCR, maximum OCR, proton leak OCR, buffered OCR, and nonmitochondrial OCR of mutant and control cell lines (^∗^*P* < 0.05).

To assess whether the MT-ND2 m. 5178C>A gene mutation affects mitochondrial respiratory functions, we calculated OCR curves of mutants and control cell lines after administration of various inhibitors. Basal OCR refers to the difference in OCR after treatment with rotenone and antimycin A before oligomycin treatment, reflecting the basic OCR of mitochondria. The basal OCR of the mutant group was increased by 42.3% compared with that of the control group (104.39 ± 8.7 vs. 60.6 ± 8.14, *P* < 0.01, Figure 4).

ATP-linked OCR refers to the difference in OCR between oligomycin treatment and posttreatment, reflecting the OCR of mitochondrial OXPHOS coupling. The ATP-linked OCR of the mutant group was increased by 37.7% compared with the control group (50.1 ± 5.65 vs. 35.6 ± 5.03, *P* < 0.01, Figure 4).

The maximum OCR value refers to the difference between the OCR when treated with FCCP and the nonmitochondrial OCR when treated with rotenone and antimycin A, reflecting the buffering capacity of mitochondrial oxygen utilization. The maximal OCR of the mutation group was increased by 50.38% compared with the control group (233.68 ± 35.5 vs. 115.94 ± 16.18, *P* < 0.01, Figure 4).

Proton leak OCR refers to the remaining OCR after treatment with rotenone and antimycin A, reflecting the rate of oxygen consumption not used by mitochondria. The proton leak OCR of the mutant group was increased by 78% compared with that of the control group (45.16 ± 5.65 vs. 9.9 ± 6.27, *P* < 0.01, Figure 4).

Reserve OCR is the difference between maximum and base OCRs, reflecting the buffering capacity of mitochondrial oxygen utilization. The reserve capacity of the mutation group was increased by 57.2% compared with that of the control group (129.41 ± 32.33 vs. 55.33 ± 16.47, *P* < 0.01, Figure 4).

Nonmitochondrial OCR refers to the remaining OCR after treatment with rotenone and antimycin A, reflecting the consumption rate of oxygen that is not used by mitochondria. The nonmitochondrial OCR of the mutant group was increased by 28.2% compared with that of the control group (17.17 ± 7.65 vs. 12.33 ± 4.68, *P* = 0.149, Figure 4).

### 2.11. Mitochondrial Complex I Activity

**Figure 5:**
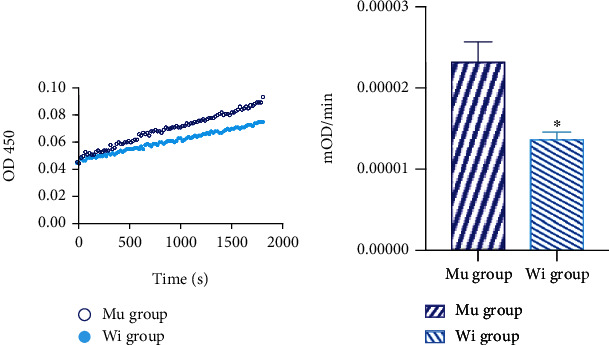
Mitochondrial Complex I activity. (a) The activity of Mitochondrial Complex I was calculated through measuring the oxidation rate of NADH. The oxidation rate of NADH was detected on OD450nm. (b) The linear growth rate represents the activity of Mitochondrial Complex I (^∗^*P* < 0.05).

To investigate whether C5178A gene mutation influences Mitochondrial Complex I function, we calculate the activity of Mitochondrial Complex I through measuring the oxidation rate of NADH. The activity of Mitochondrial Complex I of the mutant group was increased than that of the control group (2.312*e* − 005 ± 1.775*e* − 006 vs. 1.361*e* − 005 ± 6.19*e* − 007, *P* = 0.0369, Figure 5).

## 3. Discussion

Mitochondria are important organelles of eukaryotic cells, which provide more than 90% energy to cells through the electron transport chain, the main source of energy [16]. The OXPHOS complex consists of five multisubunit complexes (I–V) located on the inner mitochondrial membrane. Complex I contains seven mtDNA-encoded polypeptides, and complexes III–V contain 1, 3, and 2 mtDNA-encoded polypeptides, respectively. In the previous study, we found a MT-ND2 m. 5178C>A gene mutation in the genetic screening of 817 EH cases and 821 controls in a Chinese general population [17]. ND2 is a subunit of complex I [18]. The MT-ND2 m. 5178C>A mutation leads to replacement of leucine (Leu) with methionine (Met). In 1998, Tanaka et al. [8] first reported that MT-ND2 m. 5178C>A gene mutation was more frequent in healthy controls and centenarians, suggesting that MT-ND2 m. 5178C>A gene mutation was associated with longevity. And this conclusion was further verified in a larger sample population. Later, Raule et al. [19] carried out whole mitochondrial sequencing analysis on 2200 ultranonagenarians and controls and proved that the mutations of mtDNA can lead to the synthesis obstacle of OXPHOS complex. They found that mutations in subunits of the OXPHOS complex I had a beneficial effect on longevity. A study has shown that the substitution of methionine plays an endogenous antioxidant role in protecting mitochondria [20]. However, the specific effect of MT-ND2 m. 5178C>A mutation on OXPHOS and apoptosis is not clear.

MT-ND2 m. 5178C>A has also been reported to be associated with protective effects against various diseases. In alloxan-resistant mice, the MT-ND2 m. 4738C>A mutation is a homologous site with the human MT-ND2 m. 5178C>A mutation, which also results in replacement of Leu by Met [21], indicating that the MT-ND2 m. 5178C>A mutation is a protective factor for type 1 diabetes. In our previous study, the mutation rate of MT-ND2 m. 5178C>A in hypertensive patients was significantly lower than that in the normal control group. This study showed that ATP production of the MT-ND2 m. 5178C>A mutant group was significantly higher than that of the control group. It has been speculated that this mutation contributes to the stability of complexes of the electron transport system, less susceptibility to attack by internal and external factors, and improvement of OXPHOS and ATP synthesis. ROS produced by the mutant cell line was significantly lower than that in the control group. The improvement of mitochondrial complex I activity may be associated with prevention of the damage induced by ROS in mitochondria and other organelles. ND2 is a subunit of mitochondrial respiratory chain complex I. The complex I is the initiation of the mitochondrial respiratory chain. The action of the respiratory chain complex forms a certain proton electron gradient (mitochondrial membrane potential) between the mitochondrial inner and outer membranes. It is this gradient that drives the F0F1 ATP synthase, which promotes the synthesis of ATP [22]. This increase of ATP synthesis can further promote cell activity.

OCR is another basic indicator for mitochondrial function [23]. In this study, the mitochondrial pressure test was performed using the Seahorse XF Energy Analyzer to measure the mitochondrial OCR. Increased basal OCR, ATP-linked OCR, maximal OCR, and reserve capacity OCR reflect that the mitochondrial OCR of the mutant cells is higher than that of the control group, which was associated with improved OXPHOS. Mitochondrial membrane potential plays an important role in the maintenance of mitochondrial function and structure and participates in various diseases [24, 25]. Mitochondrial dysfunction often first shows a decrease in mitochondrial membrane potential [26]. Our previous studies have found that, in the absence of ATP or calcium overload, mitochondrial swelling leads to mitochondrial permeability transition pore (mPTP) opening, causing cytochrome C and apoptotic factor to be released from mitochondria into the cytoplasm, which activates apoptosis cascade reactions such as activation of Caspase 3/7 [27, 28]. This study showed that Caspase 3/7 produced by mutant cell lines was significantly lower than that in the control group. The early apoptosis and late apoptosis in the mutant group are significant decreased than those in the control group. The mitochondrial membrane potential is a guarantee for ATP synthesis by complex V [22, 29]. This study showed that the MT-ND2 m. 5178C>A gene mutation maintained cell stability by maintaining stability of the intracellular mitochondrial membrane potential, reducing apoptosis, and synthesizing more ATP.

## 4. Conclusions

**Figure 6:**
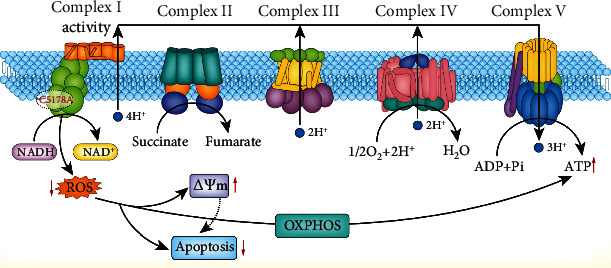
The mechanism of mitochondrial ND2 5178C>A on mitochondrial effect.

In conclusion, MT-ND2 m. 5178C>A gene mutation improves the function of mitochondrial respiratory chain complex I, reduces ROS production, inhibits the damage induced by ROS in mitochondria and other organelles, and maintains the intracellular steady state of the mitochondrial membrane potential. It also increases OCR during OXPHOS, produces more ATP, increases the cell proliferation rate, and reduces apoptosis (Figure 6). These reveal that a possible mechanism of MT-ND2 m. 5178C>A gene mutation decreased the incidence of EH and provide a new target to explore the pathogenesis of EH.

## 5. Materials and Methods

### 5.1. Study Population and Cell Lines

This study clarified the effect of MT-ND2 m. 5178C > A gene mutation on cell and mitochondrial functions. In the Chinese mitochondrial genetic screening research program [30], informed consent, blood samples, and clinical evaluations were obtained from all participating family members under protocols approved by the Ethics Committee of the Chinese PLA General Hospital Institutional Review Board. Written informed consent and consent for publication were obtained from all participants. According to the sequencing results, five individuals with the MT-ND2 m. 5178C>A gene mutation and controls were selected to establish immortalized lymphocyte lines (for more details, see reference [4]).

### 5.2. Cell Proliferation and Activity Measurements

Cell suspensions (100 *μ*L/well) were seeded in a 96-well plate at 2000, 4000, 6000, and 8000 cells/well. CCK-8 solution (CCK8 test kit, Japan) was added to each well according to the manufacturer's instructions. Plates were incubated in a cell culture incubator for 4 h and then analyzed at 450 nm using a microplate reader (BioTek, U.S.).

### 5.3. The Expression of Proliferation and Apoptosis Genes and Proteins

Cells were seeded at 2000, 4000, 6000, and 8000 cells/well, and then, 100 *μ*L Caspase-Glo ® 3/7 Reagent (Caspase-Glo ® 3/7 Assays, Promega corporation, U.S.) was added to each well. The samples were mixed gently and incubated for 3 h at room temperature. The amount of luminescence was measured in a Centro LB960 XS3 luminometer (Berthold, Germany).

Total RNA was collected by TRIzol reagent (TaKaRa, Japan) following the manufacturer's instruction. Then, RNA was reverse transcribed to cDNA by using reverse transcriptase (Promega GoTaq qPCR Master Mix, China). The sequences of Cyclin D1 primers were forward: 5′- CCCACTCCTACGATACGC-3′; reverse: 5′-AGCCTC CCAAACACCC-3′. The sequences of BCL-2 primers were forward: 5′-GATTGT GGCCTTCTTTGAGTT-3′; reverse: 5′-AGTTCCACAAAGGCATCCCA-3′. The sequences of BAX primers were forward: 5′-GGGTTGTCGCCCTTTTC TACTT-3′; reverse: 5′-TGTCCAGCCCATGATGGTTCT-3′. qRT-PCR analysis was actualized by Bio-Rad CFX96 Real-Time PCR Detection System (Bio-Rad, U.S.).

Cells were resuspended in lysis buffer (20 mM Tris pH 7.5, 2 mM EDTA, 3 mM EGTA, 2 mM dithiothreitol, 250 mM sucrose, 0.1 mM phenylmethylsulfonyl fluoride, and 1% Triton X-100) with a protease inhibitor cocktail at 4°C for 1 h. Then, the cells were centrifuged at 13,000 rpm at 4°C for 30 min. Supernatants were boiled and separated on SDS-polyacrylamide gel, then transferred to nitrocellulose membranes. The membranes were coincubated with anti Bcl-2 antibody (Santa Cruz Biotechnology, U.S.) at 4°C overnight. After washing 4 times, the membranes were incubated with secondary antibodies conjugated with horseradish peroxidase (HRP). Antigen–antibody complexes were visualized by enhanced chemiluminescence. The densitometric measurements were analyzed by using ImageJ software.

### 5.4. Apoptosis Rate Assay

The apoptosis rate was calculated through the FITC Annexin V Apoptosis Detection Kit I (BD Pharmingen, U.S.) according to the instruction from the manufacturer. The numbers of 5∗10^5^ cells were collected and washed with PBS and resuspended with 100ul 1∗ Annexin V Binding Buffer. Then, 5 *μ*L FITC Annexin V and 5 *μ*L propidium iodide were added and incubated for 15 minutes in the dark environment. Then, another 400 *μ*L 1∗ Annexin V Binding Buffer was added before being analyzed by flow cytometry (Becton Dickinson, U.S.).

### 5.5. ATP Synthesis Detection

Cells were seeded at 2000, 4000, 6000, and 8000 cells/well and incubated for 30 min at room temperature. The same amount of reagents (CellTiter-Glo Cell ATP Detection Kit, Promega Corporation, U.S.) was added to the medium per well. Cells were shaken on a shaker for 2 min to induce lysis. The cells were incubated for 10 min at room temperature to stabilize the fluorescence signals, and luminescence was recorded.

### 5.6. ROS Synthesis Assay

Fluorescent probe DCFH-DA was diluted at 1 : 1000 in RPMI 1640 medium to 10 *μ*M. Logarithmically growing cells were collected and adjusted to 1 × 10^6^/mL. The cell lines were incubated for 30 min at 37°C with 5% CO_2_ and then mixed for 3 min. The cells were washed three times with serum-free medium to remove DCFH-DA. The cell lines were harvested and analyzed for ROS by flow cytometry (Becton Dickinson, U.S.) at 488 nm excitation and 525 nm emission.

### 5.7. Mitochondrial Membrane Potential (*ΔΨ*_m_) Detection

To prepare the JC-10 dye loading solution, 50 *μ*L of 100× JC-10 was added to 5 mL Assay Buffer A. The negative control group was medium only, and the positive control group was 2 mM FCCP or CCCP. The cell lines were resuspended in 50 *μ*L/well JC-10 dye loading solution and incubated at 37°C with 5% CO_2_ while protected from light for 30 min. The fluorescence intensity was then detected in cell lines.

### 5.8. Mitochondrial OCR Assay

Cell suspensions (80 *μ*L) were added to a Cell-Tak well plate. Then, 25 *μ*L of different reagents was added to A–D while facing the sensor cartridge: A well: oligomycin (2 *μ*M); B well: FCCP (1 *μ*M); and C well: rotenone/antimycin A (0.5 *μ*M) at a final concentration of 1 *μ*M. Cellular oxygen consumption was detected by an XF96 cell energy metabolism real-time analyzer (Seahorse, U.S.) [31].

### 5.9. Mitochondrial Complex I Activity

Mitochondrial Complex I also known as NADH dehydrogenase is the largest protein complex in the inner mitochondrial membrane. Complex I can catalyze the dehydrogenation of NADH to generate NAD+. The oxidation rate of NADH is measured at 340 nm using a microplate reader (BioTek, U.S.) to calculate the size of the enzyme activity. Mitochondrial Complex I activity detection kit (mitochondrial respiratory chain complex I activity detection kit, Abcam, U.S.) was added to each well according to the manufacturer's instructions.

### 5.10. Statistical Analysis

Continuous variables are expressed as mean ± standard deviation. Nonparametric test was used for the comparison between the two groups. Statistical analyses were performed using the Statistical Package for Social Sciences software (SPSS, version 18.0). The test criteria were statistically significant at *P* < 0.05.

## Figures and Tables

**Table 1:** The clinical characteristics compared between the mutant and control groups.

Variables	mt5178C (*n* = 5)	mt5178A (*n* = 5)	*P* value
Age (years)	55.3 ± 3.2	54.6 ± 3.1	0.734
Male, *n* (%)	4 (80.0)	4 (80.0)	1
BMI (kg/m^2^)	26.2 ± 3.5	24.1 ± 2.5	0.306
Smoking, *n* (%)	3 (60.0)	3 (60.0)	1
Drinking, *n* (%)	2 (40.0)	3 (60.0)	0.655
TC (mmol/L)	4.73 ± 0.63	4.27 ± 0.42	0.214
Triglyceride (mmol/L)	1.79 ± 0.14	1.18 ± 0.43	0.016∗
LDL (mmol/L)	2.75 ± 0.36	2.51 ± 0.17	0.214
HDL (mmol/L)	1.17 ± 0.36	1.28 ± 0.23	0.581
Creatinine (*μ*mol/L)	82.91 ± 8.92	76.19 ± 9.42	0.280
BUN (*μ*mol/L)	5.29 ± 0.14	4.42 ± 0.16	0.051
SBP (mmHg)	148.00 ± 14.00	115.00 ± 7.00	0.015^∗^
DBP (mmHg)	87.00 ± 7.00	76.00 ± 4.00	0.015^∗^

BMI: body mass index; TC: total cholesterol; LDL: low-density lipoprotein; HDL: high-density lipoprotein; BUN: blood urea nitrogen; SBP: systolic blood pressure; DBP: diastolic blood pressure. ∗ indicates *P* < 0.05.

## Data Availability

The data material used and analyzed during this study is available from the corresponding author on reasonable request.

## Abbreviations

ND2: NADH dehydrogenase subunit 2
OCR: Oxygen consumption rate
mtDNA: Mitochondrial DNA
OXPHOS: Oxidative phosphorylation
rRNA: Ribosomal RNA
Leu: Leucine
Met: Methionine
*ΔΨ*_m_: Mitochondrial membrane potential
BAX: Bcl-2-associated X protein
Bcl-2: B cell lymphoma 2 protein.
